# Association between Monocyte to High-Density Lipoprotein Cholesterol Ratio and Nonalcoholic Fatty Liver Disease: A Cross-Sectional Study

**DOI:** 10.1155/2021/6642246

**Published:** 2021-12-07

**Authors:** Hangkai Huang, Qinqiu Wang, Xiaoying Shi, Yishu Chen, Chao Shen, Juanwen Zhang, Chengfu Xu

**Affiliations:** ^1^Department of Gastroenterology, The First Affiliated Hospital, Zhejiang University School of Medicine, Hangzhou 310003, China; ^2^Health Management Center, The First Affiliated Hospital, Zhejiang University School of Medicine, Hangzhou 310003, China; ^3^Department of Laboratory Medicine, The First Affiliated Hospital, Zhejiang University School of Medicine, Hangzhou 310003, China

## Abstract

**Background:**

The aim of the present study was to investigate the association between monocyte to high-density lipoprotein cholesterol ratio (MHR) and nonalcoholic fatty liver disease (NAFLD) in Chinese population.

**Methods:**

We enrolled 14189 individuals who attended their annual health examinations in the study. We performed the anthropometric and laboratory measurements and diagnosed NAFLD by hepatic ultrasonography without evidence of other etiologies of chronic liver disease. Student's *t*-test, Mann–Whitney *U* test, and chi-squared (*χ*^2^) test was used to compare the differences of clinical characteristics between participants with or without NAFLD. Pearson's and Spearman's analyses were performed to assess the correlation of MHR and NAFLD risk factors. Univariate and multivariate logistic regression analyses were conducted to explore whether MHR associated with NAFLD.

**Results:**

Thirty-five percent of the participants enrolled were diagnosed with NAFLD. Compared with healthy controls, NAFLD patients were male predominant, older, and had higher body mass index, waist circumference, and systolic and diastolic blood pressure, as well as higher levels of alanine aminotransferase, aspartate aminotransferase, *γ*-glutamyl transferase, triglyceride, total cholesterol, low-density lipoprotein cholesterol, fasting plasma glucose, glycated hemoglobin A1c, and serum uric acid, but lower levels of serum high-density lipoprotein cholesterol. Besides, MHR was significantly higher in NAFLD patients than healthy controls [5.35 (4.18–6.84) versus 4.53 (3.48–5.93), *P* < 0.001]. MHR quartiles were positively related to the prevalence of NAFLD (*P* < 0.001 for trend). In multivariate logistic regression analysis, MHR was positively associated with the risk of NAFLD after adjusting age, gender, body mass index, waist circumference, diastolic blood pressure, alanine aminotransferase, triglyceride, total cholesterol, fasting plasma glucose, and serum uric acid (OR: 1.026, 95% CI: 1.002–1.052; *P* = 0.037).

**Conclusions:**

MHR is significantly and positively associated with the risk of NAFLD.

## 1. Introduction

Nonalcoholic fatty liver disease (NAFLD) has emerged as one of the most common global health problems, affecting more than 25 percent of adults worldwide [1]. Its prevalence is expected to increase to 33.5 percent in 2030 in the United States [2]. Similarly, the prevalence of NAFLD in China has also climbed from 15 percent in the early 2000s to 29.2 percent in 2020 [3]. NAFLD can be categorized histologically into simple fatty liver, steatohepatitis, and related fibrosis and cirrhosis [4]. Recently, an increasing body of evidence showed that NAFLD patients have a markedly increased risk of hepatocellular carcinoma [5]. NAFLD is also closely associated with type 2 diabetes mellitus (T2DM), obesity, hypertension, and other components of metabolic syndrome (MetS) [6].

Potential biomarkers for noninvasive diagnosis of NAFLD are now under extensive investigation, which could be classified into blood-based, imaging, genetic, and omic biomarkers [7]. Not only do these markers noninvasively identify NAFLD patients but they also contribute to assess the severity of steatohepatitis and fibrosis. With regard to blood-based markers, including indices of apoptosis, inflammation, oxidative stress, adipokines and hormones, their values as potential diagnostic biomarkers have been examined. Chronic low-grade inflammation has been recognized as a vital part in the pathophysiology of NAFLD, suggesting that markers of chronic inflammation may predict the presence and development of NAFLD [8]. C-reactive protein, tumor necrosis factor-*α*, interleukin-6 and -8, soluble interleukin-1 receptor type 1, and monocyte chemoattractant protein 1 are indices of chronic inflammation that were confirmed to be associated with NAFLD [9, 10]. However, they were not validated as diagnostic markers for limited specificity and sensitivity [7].

Monocytes are deemed as a marker of inflammatory status as they can promote the expression of proinflammatory cytokines [11]. Besides, high-density lipoprotein cholesterol (HDL-C) exhibits antioxidant and anti-inflammatory effects in many pathological conditions including diabetes mellitus, atherosclerosis, and chronic kidney disease [12]. Given the proinflammatory properties of monocytes and the anti-inflammatory properties of HDL-C, monocyte to HDL-C ratio (MHR) has gradually been viewed as a novel biomarker of systemic inflammation [13]. Elevated MHR value has been reported as an independent predictor of poor outcomes in patients with suspected stable coronary artery disease [13] and with acute coronary syndrome [14]. However, whether MHR is associated with NAFLD remains unknown.

In this study, we conducted a large cross-sectional study to estimate the association between MHR and NAFLD and to explore whether MHR could act as a novel and practical biomarker for noninvasive diagnosis of NAFLD.

## 2. Materials and Methods

### 2.1. Study Population

**Figure 1:**
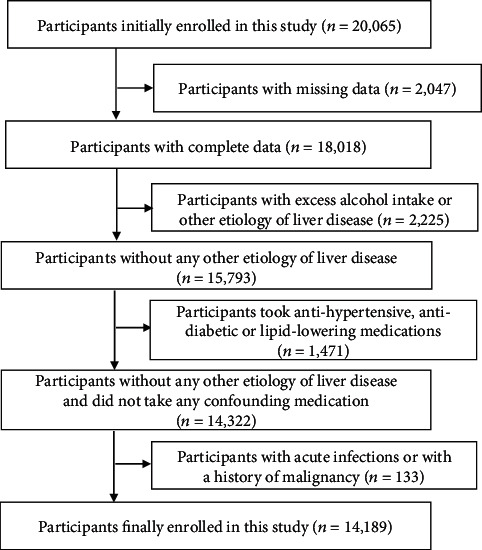
Flowchart of the study.

The study population was recruited from adults who attended their annual health examinations at the First Affiliated Hospital, Zhejiang University School of Medicine in 2014. Exclusion criteria were as follows: (i) those with incomplete data; (ii) those with excess alcohol intake or any evidence of other etiologies of chronic liver disease such as drug-induced liver disease and viral hepatitis; (iii) those self-reportedly under antihypertensive, antidiabetic, or lipid-lowering medications; and (iv) those with acute infections within 2 weeks or with a history of malignancy. A total of 14189 participants were enrolled in this study for analysis (Figure 1). The ethical approval of this study was obtained from the Ethic Committee of the First Affiliated Hospital, Zhejiang University School of Medicine.

### 2.2. Clinical Examinations

Anthropometric and biochemical measurements were conducted as previously described [15–17]. Both height and body weight were measured with shoes taken off in light clothes. Waist circumference was taken with a nonretractable tape at one centimeter above the umbilicus. Blood pressure was determined by an automatic sphygmomanometer at a resting state (staying still for five minutes).

All participants were fasted overnight before taking laboratory tests. Venous blood samples were obtained for measurements of liver enzymes, glucose, serum lipids, uric acid ,and monocyte counts with a Hitachi 7600 autoanalyzer (Hitachi, Tokyo, Japan) or a Sysmex XE-2100 auto-analyzer (Sysmex, Kobe, Japan). MHR was calculated as monocyte counts (10^9^/L) divided by HDL-C (mmol/L).

### 2.3. Diagnosis of NAFLD

NAFLD was diagnosed according to the criteria suggested by the Chinese Liver Disease Association, based on ultrasonographical presentation of fatty liver to the exclusion of other etiologies of chronic liver disease. The presence of at least two of the three findings below was defined as fatty liver: (i) “bright liver,” (ii) liver echo greater than kidney; and (iii) vascular blurring and the gradual attenuation of far field ultrasound echo [18]. Experienced ultrasonographists who were blind to the research design conducted the abdominal ultrasonography with a 3.5 MHz transducer (Siemens, Erlangen, Germany).

### 2.4. Statistical Analysis

Continuous variables were described as mean and standard deviation if normally distributed or otherwise as median and interquartile range (IQR). Comparisons of anthropometric and laboratory parameters were performed using Student's *t*-test, Mann–Whitney *U* test, and chi-squared (*χ*^2^) test as needed. Pearson's and Spearman's analyses were conducted to examine the correlations between MHR and metabolic parameters. Binary univariate and multivariate logistic regression analyses (backward: Wald; cutoff for entry: 0.05, for removal: 0.10) were used to determine risk factors for NAFLD. We first identified potential risk factors associated with NAFLD in the backward stepwise univariate logistic regression analysis. These variables were then subject to the initial equation of backward stepwise multivariate logistic regression analysis. Variables with statistical significance were reserved in the equation. These calculations were performed by SPSS 20.0 (SPSS Inc., Chicago, IL). A *P* value smaller than 0.05 (two-tailed) was considered statistically significant.

## 3. Result

### 3.1. Clinical Characteristics of the Participants

Of the 14189 participants, 4965 (35.0%) were diagnosed with NAFLD. We compared clinical profiles of these participants on the basis of NAFLD status (Table 1). We found that NAFLD patients were male predominant, older and had higher body mass index, waist circumference, and systolic and diastolic blood pressure, as well as higher levels of alanine aminotransferase, aspartate aminotransferase, *γ*-glutamyl transferase, triglyceride, total cholesterol, low-density lipoprotein cholesterol, fasting plasma glucose, glycated hemoglobin A1c, and serum uric acid, but lower levels of serum HDL-C, than healthy controls. These results suggested that NAFLD patients had less desirable metabolic profiles than controls. Of note, we observed that NAFLD patients had markedly higher MHR than healthy controls [5.35 (4.18–6.84) versus 4.53 (3.48–5.93), *P* < 0.001; Table 1].

### 3.2. Association between MHR and Prevalence of NAFLD

We divided all the participants into quartiles based on their MHR levels: ≤3.70, 3.71–4.81, 4.82–6.25, and ≥6.26. We observed a positive relationship between MHR quartiles and NAFLD prevalence, which was 21.9% in the first quartile, and increased to 31.9%, 39.7%, and 46.6% in the second, third, and fourth quartile, respectively (Table 2). These results indicated that participants with high MHR values were much more likely to have NAFLD than those with low MHR values.

### 3.3. Association between MHR and NAFLD-Related Metabolic Profiles

We conducted linear correlation analysis to investigate the correlations between MHR and NAFLD-related metabolic profiles. We observed that MHR was inversely correlated with age, body mass index, waist circumference, systolic and diastolic blood pressure, alanine aminotransferase, aspartate aminotransferase, *γ*-glutamyl transferase, triglyceride, fasting plasma glucose, and glycated hemoglobin A1c (Table 3). These results suggested a close association between MHR and NAFLD-related metabolic profiles.

### 3.4. Association between MHR and Risk of NAFLD

We further performed univariate and multivariate logistic regression analyses to explore whether MHR was significantly related to the risk of NAFLD. In the univariate model, we found that age, gender (male), body mass index, waist circumference, systolic and diastolic blood pressure, alanine aminotransferase, aspartate aminotransferase, *γ*-glutamyl transferase, triglyceride, total cholesterol, low-density lipoprotein cholesterol, fasting plasma glucose, glycated hemoglobin A1c, and serum uric acid were positively, while HDL-C was inversely, associated with NAFLD prevalence. Besides, elevated MHR value was significantly associated with the risk of NAFLD (OR: 1.193, 95% CI: 1.173–1.213; *P* < 0.001) (Table 4).

Considering that alanine aminotransferase, aspartate aminotransferase, and *γ*-glutamyl transferase are all markers of liver injury, LDL-C and HDL-C are both components of total cholesterol, fasting plasma glucose and HbA1c are both indicators of T2DM, and we included age, gender, BMI, waist circumference, systolic and diastolic blood pressure, alanine aminotransferase, triglyceride, total cholesterol, fasting plasma glucose, serum uric acid, and MHR in to the multiple logistic regression analysis. After adjustment of these cofounders, MHR remained markedly associated with an increased risk of NAFLD (OR: 1.026, 95% CI: 1.002–1.052; *P* = 0.037) (Table 5).

## 4. Discussion

In this large cross-sectional study, we observed that MHR was positively associated with NAFLD. First, NAFLD patients had significantly higher MHR values than healthy controls. Second, MHR was positively related to NAFLD prevalence. Third, MHR was independently associated with an increased risk of NAFLD.

Circulating monocytes in humans can be classified into three subgroups in light of surface expressions of CD14 and CD16. The classical monocytes (CD14^++^CD16^−^) make up ≥92% of monocytes in the peripheral blood and play a proinflammatory role via releasing interleukin-8 and tumor necrosis factor-*α* (TNF-*α*) upon lipopolysaccharide stimuli. The nonclassical monocytes (CD14^+^CD16^++^) have been reported to be involved in the production of interleukin-1*β* and TNF-*α*, though still under controversy. The intermediate monocytes (CD14^++^CD16^+^) have been found to release anti-inflammatory interleukin-10 as well as proinflammatory cytokines like interleukin-1*β* and TNF-*α* [19, 20]. In contrast, HDL-C exhibits anti-inflammatory effects. Cockerill et al. [21] observed that the expression of E-selectin induced by interleukin-1 in acute inflammation could be suppressed by increasing plasma HDL. Interestingly, HDL was reported to suppress the expression of monocyte chemotactic protein 1, which plays a crucial role in monocyte migration [22]. Taking into account the opposite effects of monocytes and HDL-C, more and more studies suggested that MHR could act as a novel and cost-effective marker of inflammation, especially in cardiovascular events [23].

Previous studies have demonstrated that MHR was an independent predictor of the presence and prognosis of cardiovascular diseases (CVD). Elevated MHR was positively related to the presence of cardiac syndrome X, which was associated with poor cardiovascular outcomes [24]. Besides, in patients undergoing coronary angiography, higher MHR levels were linked to increased major adverse cardiac events and decreased event-free survival [13]. In patients with ST-segment elevation myocardial infarction after percutaneous coronary intervention, increased MHR values were observed to be positively associated with stent thrombosis [14], no reflow [25], and inhospital and long-term mortality [26]. Similarly, MHR was found to be a novel predictor of metabolic disorders including metabolic syndrome [27] and polycystic ovary syndrome [28]. In view of the strong association between NAFLD and CVD [29], polycystic ovary syndrome [30], and metabolic syndrome [31], we hypothesized that MHR could also act as a predictive marker for NAFLD.

Previous studies have revealed that monocyte infiltration was increased in NASH models compared with that in controls, and hepatic inflammation and fibrosis could be alleviated by pharmacological suppression of monocyte recruitment [32]. In addition, the role of non-HDL-C in predicting the risk of incident NAFLD was verified in a seven-year follow-up study [33]. Considering the fact that monocyte or non-HDL-C alone could serve as a NAFLD biomarker, we investigated whether MHR, a combination of these two indicators, could perform better in identifying NAFLD patients. We found that MHR was superior to monocyte for NAFLD diagnosis in all participants and to non-HDL-C in individuals aged >65 years. However, MHR was not superior to non-HDL-C among all participants. Further prospective studies are needed to explore whether MHR is superior to monocyte or non-HDL-C alone to predict the outcomes among patients with NAFLD.

As mentioned above, MHR has gradually been viewed as a novel marker of inflammation, and thus we speculate that the underlying mechanism linking MHR to NAFLD may possibly fall on inflammation. As a biomarker of systemic inflammation, C-reactive protein was observed to be positively correlated with MHR values in various cardiovascular events, including stable and unstable coronary artery disease [34, 35]. Hepatic macrophages, which contain monocyte-derived macrophages and Kupffer cells, are involved in NAFLD progression and liver fibrosis [36]. After activation, hepatic macrophages release proinflammatory interleukin-1*β* and TNF-*α*, which facilitate the inflammatory transition from simple fatty liver to steatohepatitis [37, 38]. Moreover, monocyte-derived macrophages were reported to promote liver cirrhosis via producing transforming growth factor-*β* and platelet-derived growth factor [39]. On the other hand, HDL-C exhibits antioxidative effects against monocytes via suppressing the generation of oxidized low-density lipoprotein cholesterol and activation and proliferation of monocytes, especially in its atheroprotective role [40]. Oxidative stress is believed to play a vital role in the pathogenesis of NAFLD.

Our study has several limitations. First, circulating monocytes can be classified into three subsets which play different roles in inflammation. Our study mainly focused on total monocyte counts without distinguishing between subsets. Second, given that this was a cross-sectional study, we failed to assess the causality of the relationship between MHR and NAFLD. Third, we did not detect markers of systemic inflammation like TNF-*α* and interleukin-6 or markers of oxidative stress like serum superoxide dismutase. Fourth, NAFLD was diagnosed by abdominal ultrasonography, which is not sensitive in detecting mild hepatic steatosis or further progressive stages such as steatohepatitis or fibrosis. Consequently, the association of MHR values with pathological changes in NAFLD could not be determined in this study. These issues need to be clarified in further studies.

In conclusion, our large cross-sectional study showed that MHR, a novel and practical biomarker of systemic inflammation, is independently and positively associated with NAFLD.

## Figures and Tables

**Table 1:** Comparison of clinical characteristics between the subjects with and without NAFLD.

Variables	With NAFLD (*n* = 4965)	Without NAFLD (*n* = 9224)	*Z* value	*P* value
Age (year)	52 (45–59)	47 (40–55)	-19.838	<0.001
Gender (male/female, *n*)	3683/1282	4565/4659	808.317†	<0.001
BMI (kg/m^2^)	25.96 (24.34–27.77)	22.39 (20.65–24.26)	-65.179	<0.001
Waist circumference (cm)	91 (86–96)	80 (74–86)	-61.779	<0.001
Systolic blood pressure (mmHg)	133 (122–144)	121 (111–133)	-36.960	<0.001
Diastolic blood pressure (mmHg)	82 (75–89)	74 (67–82)	-38.525	<0.001
Alanine aminotransferase (U/L)	26 (18–37)	16 (12–22)	-48.523	<0.001
Aspartate aminotransferase (U/L)	22 (18–27)	19 (16–23)	-29.232	<0.001
*γ*-Glutamyl transferase (U/L)	34 (22–55)	17 (12–27)	-50.255	<0.001
Triglyceride (mmol/L)	1.72 (1.24–2.46)	1.00 (0.73–1.41)	-54.937	<0.001
Total cholesterol (mmol/L)	4.94 (4.36–5.58)	4.59 (4.07–5.17)	-21.749	<0.001
HDL-C (mmol/L)	1.10 (0.95–1.30)	1.32 (1.12–1.55)	-38.557	<0.001
LDL-C (mmol/L)	2.70 (2.28–3.16)	2.51 (2.12–2.95)	-15.274	<0.001
Fasting plasma glucose (mmol/L)	5.03 (4.70–5.55)	4.75 (4.49–5.05)	-32.586	<0.001
HbA1c (%)	7.00 (6.70–7.50)	6.70 (6.50–7.00)	-28.698	<0.001
Serum uric acid (*μ*mol/L)	369 (313–430)	298 (246–358)	-42.845	<0.001
MHR	5.35 (4.18–6.84)	4.53 (3.48–5.93)	-23.423	<0.001

Data are presented as median (IQR) due to skewed distribution. ^†^*χ*^2^ value; BMI: body mass index; HbA1c: glycated hemoglobin A1c; HDL-C: high-density lipoprotein cholesterol; LDL-C: low-density lipoprotein cholesterol; MHR: monocyte to HDL-C ratio; NAFLD: nonalcoholic fatty liver disease.

**Table 2:** Association of MHR with prevalence rate of NAFLD.

MHR quartiles	Total	NAFLD	PR%	PR	*χ* ^2^	*P* value
Quartile 1	3560	780	21.9%	1.00	525.126	<0.001
Quartile 2	3521	1122	31.9%	1.46		
Quartile 3	3568	1415	39.7%	1.81		
Quartile 4	3540	1648	46.6%	2.13		

MHR: monocyte to HDL-C ratio; NAFLD: nonalcoholic fatty liver disease; PR%: prevalence rate; PR: prevalence ratio.

**Table 3:** Correlations between MHR and metabolic parameters.

	Age	BMI	WC	SBP	DBP	ALT	AST	GGT	TG	FPG	HbA1c
*r* value	0.024	0.263	0.307	0.111	0.118	0.230	0.132	0.243	0.289	0.047	0.105
*P* value	0.004	<0.001	<0.001	<0.001	<0.001	<0.001	<0.001	<0.001	<0.001	<0.001	<0.001

ALT: alanine aminotransferase; AST: aspartate aminotransferase; BMI: body mass index; DBP: diastolic blood pressure; FPG: fasting plasma glucose; GGT: *γ*-glutamyl transferase; HbA1c: glycated hemoglobin A1c; SBP: systolic blood pressure; TG: triglyceride; WC: waist circumference.

**Table 4:** Univariable analysis for factors associated with NAFLD.

Variables	*β*	SE	Wald *χ*^2^	*P* value	OR	95% CI
Gender (male/female)	1.076	0.039	779.067	<0.001	2.932	2.719–3.162
Age (year)	0.026	0.002	305.080	<0.001	1.027	1.024–1.030
BMI (kg/m^2^)	0.453	0.009	2680.124	<0.001	1.573	1.546–1.600
Waist circumference (cm)	0.164	0.003	2635.793	<0.001	1.178	1.171–1.186
Systolic blood pressure (mmHg)	0.039	0.001	1158.020	<0.001	1.040	1.037–1.042
Diastolic blood pressure (mmHg)	0.064	0.002	1259.274	<0.001	1.066	1.062–1.070
Alanine aminotransferase (U/L)	0.045	0.001	999.223	<0.001	1.046	1.043–1.048
Aspartate aminotransferase (U/L)	0.037	0.002	262.613	<0.001	1.038	1.033–1.042
*γ*-Glutamyl transferase (U/L)	0.017	0.001	674.271	<0.001	1.017	1.016–1.019
Triglyceride (mmol/L)	1.045	0.026	1587.221	<0.001	2.844	2.701–2.994
Total cholesterol (mmol/L)	0.437	0.020	461.541	<0.001	1.548	1.488–1.611
HDL-C (mmol/L)	-2.418	0.069	1241.847	<0.001	0.089	0.078–0.102
LDL-C (mmol/L)	0.378	0.027	201.467	<0.001	1.459	1.385–1.538
Fasting plasma glucose (mmol/L)	0.613	0.025	590.743	<0.001	1.845	1.756–1.939
HbA1c (%)	0.784	0.037	444.916	<0.001	2.191	2.037–2.357
Serum uric acid (*μ*mol/L)	0.009	0.000	1563.673	<0.001	1.009	1.009–1.010
MHR	0.177	0.009	424.782	<0.001	1.193	1.173–1.213

BMI: body mass index; *β*: partial regression coefficient; HbA1c: glycated hemoglobin A1c; HDL-C: high-density lipoprotein cholesterol; LDL-C: low-density lipoprotein cholesterol; MHR: monocyte to HDL-C ratio; NAFLD: nonalcoholic fatty liver disease; SE: standard error of partial regression coefficient.

**Table 5:** Multivariable analysis for factors associated with NAFLD.

Variables	*β*	SE	Wald *χ*^2^	*P* value	OR	95% CI
Gender (male/female)	0.611	0.072	72.993	<0.001	1.843	1.602–2.121
Age (year)	0.006	0.002	5.921	0.015	1.006	1.001–1.011
BMI (kg/m^2^)	0.228	0.016	214.860	<0.001	1.256	1.218–1.295
Waist circumference (cm)	0.070	0.006	153.006	<0.001	1.073	1.061–1.085
Diastolic blood pressure (mmHg)	0.013	0.002	28.945	<0.001	1.013	1.008–1.018
Alanine aminotransferase (U/L)	0.012	0.002	66.098	<0.001	1.012	1.009–1.015
Triglyceride (mmol/L)	0.428	0.030	202.356	<0.001	1.535	1.447–1.628
Total cholesterol (mmol/L)	0.100	0.031	10.742	0.001	1.105	1.041–1.174
Fasting plasma glucose (mmol/L)	0.264	0.025	108.257	<0.001	1.302	1.239–1.368
Serum uric acid (*μ*mol/L)	0.004	0.000	114.931	<0.001	1.004	1.003–1.005
MHR	0.026	0.012	4.357	0.037	1.026	1.002–1.052

BMI: body mass index; *β*: partial regression coefficient; LDL-C: low-density lipoprotein cholesterol; MHR: monocyte to HDL-C ratio; NAFLD: nonalcoholic fatty liver disease; SE: standard error of partial regression coefficient.

## Data Availability

The data used to support the findings of this study are available from the corresponding author upon request.
